# The undeclared use of third-party service providers in academic publishing is unethical: an epistemic reflection and scoping review

**DOI:** 10.1007/s00210-024-03177-6

**Published:** 2024-07-11

**Authors:** Jaime A. Teixeira da Silva, Timothy Daly, Jens C. Türp, Bernhard A. Sabel, Graham Kendall

**Affiliations:** 1 Miki-cho, Japan; 2 Bioethics Program, FLACSO Argentina, Buenos Aires, Argentina; 3 Science Norms Democracy, UMR 8011, Sorbonne Université, Paris, France; 4 Department of Oral Health & Medicine, University Center for Dental Medicine UZB, University of Basel, Basel, Switzerland; 5 Institute of Medical Psychology, Medical Faculty, Otto-von-Guericke University of Magdeburg, Leipziger Straße 44, Magdeburg, 39120 Germany; 6 School of Engineering and Computing, MILA University, No. 1, Persiaran MIU, 71800 Putra Nilai, Negeri Sembilan Darul Khusus, Malaysia; 7 School of Computer Science, The University of Nottingham, University Park, Nottingham, NG7 2RD United Kingdom

**Keywords:** English, Ethics, Language editing, Outsourcing, Support, Translation, Unethical behavior

## Abstract

There is a substantial body of scientific literature on the use of third-party services (TPS) by academics to assist as “publication consultants” in scholarly publishing. TPS provide a wide range of scholarly services to research teams that lack the equipment, skills, motivation, or time to produce a paper without external assistance. While services such as language editing, statistical support, or graphic design are common and often legitimate, some TPS also provide illegitimate services and send unsolicited e-mails (spam) to academics offering these services. Such illegitimate types of TPS have the potential to threaten the integrity of the peer-reviewed scientific literature. In extreme cases, for-profit agencies known as “paper mills” even offer fake scientific publications or authorship slots for sale. The use of such illegitimate services as well as the failure to acknowledge their use is an ethical violation in academic publishing, while the failure to declare support for a TPS can be considered a form of contract fraud. We discuss some literature on TPS, highlight services currently offered by ten of the largest commercial publishers and expect authors to be transparent about the use of these services in their publications. From an ethical/moral (i.e., non-commercial) point of view, it is the responsibility of editors, journals, and publishers, and it should be in their best interest to ensure that illegitimate TPS are identified and prohibited, while publisher-employed TPS should be properly disclosed in their publications.

## Introduction

In the modern era of commercial publishing, a parallel universe of cottage industries has evolved alongside it to provide the publishing infrastructure to help authors produce publishable manuscripts with reliable content and structure (Price 2015). These third-party services (TPS) provide various types of support to researchers and authors, either to complement or optimize the skills these scientists already possess or to perform tasks they do not have the skills or time to perform or complete themselves. Other motivations for using or hiring TPS include “outsourcing” to reduce workload or to increase personal or leisure time (Benderly 2016).

Several decades ago (e.g., in the 1980s and 1990s), authors had to send physical copies of their papers by ordinary mail (i.e., post) to journals, typically in triplicate, which were then forwarded to at least two reviewers, who returned their reviews, and those were then sent back to the authors. After making the suggested revisions, the authors would mail copies of the edited paper, along with their responses to the reviewers, to the editorial office (Buchsbaum 2019). Compared to today’s standards, the process was slow, taking weeks or months, with processing speed depending on the state of technological development (typewriters, personal computers, floppy disks, faxes, e-mail attachments, etc.). The process only allowed authors to publish one or two, or at most a handful of papers, a year. With the advent of the Internet, the entire process became simplified (Kohane and Altman 2000; Chew et al. 2004) and accelerated immensely by allowing electronic communication, both in terms of e-mail and document exchange. In our experience, prior to the early 2000s, language editing by the publisher was a typical and integral part of the manuscript proofing stage, at least for elite international journals. However, the high cost of this service limited the number of scholars who could participate in the academic publishing process, essentially making this activity very exclusive (and thus exclusionary). The liberalization of academic publishing arose not only due to easier communication facilitated by the Internet and e-mail, and to some extent the advent of the open access movement, but also due to a transition from print to digital (and hence the rise of the open access movement). During that transition, some journals and publishers began to cut costs and corners, including in editing and proofreading, spurring the growth of this parallel universe of the TPS cottage industry. The digitization of academic publishing and decentralization of quality control (e.g., editing, peer review) thus also allowed a range of quality across journals and publishers to develop. In some cases, this may have also facilitated dishonest authors, as well as dishonest TPS, to populate the publishing landscape.

TPS offer a wide range of services at all educational levels, usually in the form of support in the context of school or higher education. They offer services to complete exams or essays, which constitute a special form of contract cheating (Ellis et al. 2018; Heriyati et al. 2023; Sweeney 2023; Xu and Li 2023). In this paper, we propose the ethical premise that both contract cheating and the non-disclosure of TPS (particularly those that provide intellectual content) are equivalent. Our logic for this premise is that any assistance that affects the intellectual content of a paper should be openly and transparently declared by authors. We are *not* suggesting here that authors should refrain from using TPS. However, we posit that the *non-disclosure* of the use of TPS is unethical because it represents the submission of scientific articles without full disclosure of the various contributing entities. Failure to acknowledge this assistance, through recklessness, neglect, or intentional deception, can be considered a breach of contract. Our logic is based on the general principle that there should be no intellectual contribution to a scientific article without recognition, which is based on the rights, ethics, and responsibilities of intellectual contribution to published scientific papers.

This scoping review has four objectives:

(i) To review the indexed literature on the use of TPS (primarily language services)
(ii) To assess the ethical issues associated with the undeclared use of TPS
(iii) To examine the language- and editing-related TPS offered by the ten largest commercial publishers, according to Nishikawa-Pacher (2022) (Table 1)
(iv) To reflect on the declared or undeclared use of such services

**Table 1:** Language editing services associated with the 10 largest academic publishers in the world^1^

Publisher	Name of editing service	Related URLs^2^	Archived URL^3^
Brill Academic Publishers	Provided by Academic Language Experts (in partnership with Brill)	https://www.aclang.com/services/?partner=brill	https://web.archive.org/web/20230928093630/https://www.aclang.com/services/?partner=brill
Cambridge University Press	Cambridge Author Services	https://www.cambridge.org/universitypress/author-services/	https://web.archive.org/web/20230928094029/https://www.cambridge.org/universitypress/author-services/
De Gruyter	English Language Editing Service	As one example: https://www.degruyter.com/journal/key/almed/html (see “Submission Checklist”)	Cannot be archived (downloadable PDF)
Elsevier	Elsevier Language Editing services	https://webshop.elsevier.com/language-editing/	https://web.archive.org/web/20230928091935/https://webshop.elsevier.com/language-editing/
Inderscience	None found	–	–
Oxford University Press	Oxford Academic Language Services (in partnership with Enago)	https://academic.oup.com/pages/authoring/journals/preparing_your_manuscript/language_services	https://web.archive.org/web/20230928093134/https://academic.oup.com/pages/authoring/journals/preparing_your_manuscript/language_services
SAGE	Sage Author Services	https://languageservices.sagepub.com/en/	https://web.archive.org/web/20230928092429/https://languageservices.sagepub.com/en/
Springer Nature	American Journal Experts^4^	https://authorservices.springernature.com/	https://web.archive.org/web/20230928091258/https://authorservices.springernature.com/
Taylor & Francis	Editing Services	https://tandfeditingservices.com/	https://web.archive.org/web/20230928091619/https://tandfeditingservices.com/
Wiley	Wiley Editing Services	https://wileyeditingservices.com/en/article-preparation/	https://web.archive.org/web/20230928092216/https://wileyeditingservices.com/en/article-preparation/

^1^According to Nishikawa-Pacher (2022)

^2^Accessed 5 May 2024

^3^Archived 5 May 2024

^4^Purchased by Springer Nature, together with the preprint server Research Square, in December 2022: https://group.springernature.com/jp/group/media/press-releases/springer-nature-completes-acquisition-of-research-square-company/23768186

Disclaimer: The contents of the table do not imply or suggest, either explicitly or implicitly, that the editing service organization (TPS) is providing illegitimate, fraudulent, or otherwise questionable content to its customers. These are merely examples of how publishers collaborate with outside service providers

## Background

Authors of scientific publications have both rights (Al-Khatib and Teixeira da Silva 2017) and responsibilities (Teixeira da Silva et al. 2013). Authorship has acquired an individualistic connotation in modern publishing based on the “romantic” idea of an author as someone who shapes reality “through the operation of their unique, individual genius” (Jaszi and Woodmanse 2013). However, this self-contained view of authorship is at odds with the well-recognized fact that most of the time science is a group effort within a social context (Weaver 2019). While intellectual property laws—a discussion of which is beyond the scope of this paper—are based on the individualistic view, the current widespread use of TPS demonstrates the permeability of intellectual contributions within academia. Analogous to authors, providers of TPS have their own rights and responsibilities. We therefore propose that declaring the use of TPS is a necessary condition for them to be able to exercise their rights and responsibilities within the scholarly publishing ecosystem.

This principle extends to non-commercial intellectual or technical assistance from a third party, which should always be declared, such as text editing, use of equipment, proofreading, oral discussions with colleagues, translations, creation of graphs or figures. Such declarations are typically referred to as “Acknowledgements” in academic papers (Teixeira da Silva et al. 2023). Conversely, we believe that a declaration would not be expected for minor support, where the intellectual contribution is minimal, i.e., contributions that have not reached a level where rights and responsibilities can reasonably be claimed (e.g., lending minor reagents, offering quick opinions on papers, minor edits to manuscripts, providing funding).

To illustrate our discussion, consider the following theoretical example of a language-based TPS and why there is a dual ethical obligation behind such statements. An author writes a single-author paper, but engages someone to edit the paper *extensively*, add new content and logic, and polish the English. This assistance may be provided by a colleague (non-commercial TPS) or as paid assistance provided by an external consultant, company, or publisher (i.e., a commercial TPS). If any of these services, whether commercial or non-commercial, provides input that is intellectual in nature, i.e., contributes to improved communication of the data or ideas presented, a more solid intellectual foundation, or a sounder methodological premise of a paper, we propose that authors should be required to declare such assistance in order to truthfully acknowledge the origins of the intellectual contribution of their manuscript. This is their first ethical duty.

The second ethical duty is based on the potential positive and negative consequences of publishing a paper. For instance, in the absence of a declaration, a potential employer, promotion panel, grant committee, or funding agency, to name just the most important entities, might believe that the paper was written in whole or in part by that author and might employ, fund, or promote her or him (in part) on the basis of that false assumption. The problem becomes increasingly relevant when the lack of disclosure is scaled up, i.e., the “unjust” rewards may not be noticeable with just one paper, but if several papers published over the course of a year engage TPS without declaring their use, then the improper (actually, unethical, as we argue in more detail later) and unscholarly behavior becomes amplified. If, even after several years, an author, or a group of authors, is found to have received benefits (employment, salary raises, funding, etc.) based on missing or false disclosures in their academic papers or grant proposals (in this case, the failure to declare the use of TPS), then this would be tantamount to improper gain (i.e., fraud, dishonest/false claims or statements) and could be considered gross misconduct. Conversely, if the paper is subject to serious post-publication criticism, such as misuse of technical language (e.g., falsification of data, exaggerated claims), or if such a paper is retracted from the permanent scientific record, then the TPS should also be held accountable for their intellectual contributions to such outcomes, and their professional competence should be reconsidered.

## A call for change

Here, we propose that a paper should publically declare to contain “substantial intellectual input” from third parties, if one or both of the following two conditions are met:

(i) There was assistance on a preprint^1^ or peer-reviewed paper.
(ii) Those who read the paper would do so without the full knowledge of who wrote or edited the paper.

It may seem a trivial issue not to acknowledge editing assistance, but where are the lower limits? Moreover, small initial violation can lead to more violations, one small step at a time, each of which may seem like minor infringements, but when taken together, they are actually quite serious.

In academic publishing, the most obvious language-associated TPS are related to editing services—predominantly in English, which is the *lingua franca* of scholarly publishing (Friedbichler et al. 2008). These may be provided by academic writing centers (English language institutes) or translation services, again predominantly into English, in order to be able to publish in high-impact journals that are typically associated with publication in English (Uysal and Selvi 2021). We are not suggesting that the English language is—or should be—the exclusive *lingua franca* of academic publishing or scholarly communication in all areas of learned societies. Indeed, the use of national languages is essential for certain subjects (e.g., arts, history, sociology, philosophy, medicine), and indeed, there are many journals in several non-English languages published by some of the top scholarly publishing houses in terms of journal volume (Nishikawa-Pacher 2022) (Table 1). We note that while these publishers offer language and editing services, it is not clear whether these services are provided by in-house employees or outsourced to TPS (i.e., independent companies contracted by publishers). Thus, a detailed determination and an in-depth analysis would be required in the future to clarify this issue.

Zakaria (2022) found that, among seven commercial publishers analyzed, Emerald Publishing Services had the most (12) online editing author services, provided by the Charlesworth Group, Elsevier had 10, Hindawi (part of Wiley, but debranded in December 2023) and Taylor & Francis had nine each, Springer Nature and Wiley-Blackwell had eight each, while SAGE had seven. However, most (93.3%) were provided by Enago, which includes a total of nine editing companies (American Journal Experts, Cambridge Language Consultants, Edanz, Editage Enago, ManuscriptEdit, OnLine English, Sirius Interactive, and Write Science Right) (Zakaria 2022).

To simplify the debate, when we refer to language or editing services, we are referring to services that provide assistance with English grammar, such as editing and proofreading, or the translation from any language into English which are not typically considered as intellectual contributions. In all cases, these are TPS.

To dispel a misconception early on in this paper, we note that just because a TPS may be associated with a famous brand or publisher does not imply that its use is being ethically and transparently declared. As we discuss later, truthfulness about the use of TPS is the authors’ responsibility, although editors, journals, and publishers have an equal responsibility to try to detect unethical actions^2^. If there is an error in the paper, the authors may claim that it was introduced during editing and try to absolve themselves of responsibility. However, we suspect that a porous (or non-existent) declaration, in addition to inadequate fraud detection systems by editors, journals, and publishers, may contribute to a potentially high volume of papers with ethical violations related to the lack of honest declarations about the use of TPS.

## Literature on TPS

During their research and the subsequent steps leading up to the publication of their findings in academic journals, authors may need to rely on TPS or publication consultants (Kendall et al. 2016). These may be individuals or teams within these TPS, or the TPS themselves, which can range from one-person operations to multinational organizations with hundreds or thousands of employees or consultants, offering services ranging from trivial to highly technical tasks, including integrity inspectors^3^ (Abbott 2019). In fact, they can offer services in different areas (see Table 2 for a non-exhaustive list with corresponding duties for authors, editors, and publishers). These could include either ethically acceptable services (the first five items) or unacceptable (unethical, fraudulent) services (the last three items):

- General assistance (e.g., journal selection, manuscript layout, reference formatting)
- Language (e.g., editing, proofreading, grammatical/typographical assistance, translation)
- Data generation and analysis (e.g., statistical analysis, data collection)
- Ethics (e.g., plagiarism detection, but also reducing text similarity to an acceptable level)
- Paper writing (e.g., writing a paper from a thesis)
- Data “cleaning” or fabrication
- Providing papers with people listed as authors who did not contribute
- Production of fake-papers (full service by “paper mills”: authorship trading, intentional plagiarism, fabrication of fake data and graphs, communication with journal editors on behalf of authors, etc.)

**Table 2:** Suggestions regarding the duties of the three main agents (authors, editors, publishers) regarding the use of TPS and related statements in academic papers^1^

Service	Author duties	Editor duties	Publisher duties
General assistance (e.g., journal selection, manuscript layout, reference formatting)	In their correspondence with editors, authors should declare use of such services. Authors could declare the use of such services in their manuscript’s acknowledgments section.	Editors do not have any specific duties regarding this service beyond regular quality control and conformity to submission guidelines.	Publishers should provide a list of best practices for editors and authors regarding the use of this service.
Language (e.g., editing, proofreading, grammatical/typographical assistance, translation)	In their correspondence with editors, authors should declare use of such services. Authors could declare the use of such services in their manuscript’s acknowledgments section.	In their correspondence with authors, editors should remind authors of their duty to declare the use of such services.	Publishers should provide a list of best practices for editors and authors regarding the use of this service.
Data generation and analysis (e.g., statistical analysis, data collection)	In their correspondence with editors, authors should declare use of such services. Authors could declare the use of such services in their manuscript’s acknowledgments section.	In their correspondence with authors, editors should remind authors of their duty to declare the use of such services.	Publishers should provide a list of best practices for editors and authors regarding the use of this service.
Ethics (e.g., plagiarism detection, but also reducing text similarity to an acceptable level)	In their correspondence with editors, authors should declare use of such services. Authors could declare the use of such services in their manuscript’s acknowledgments section.	In their correspondence with authors, editors should remind authors of their duty to declare the use of such services.	Publishers should provide a list of best practices for editors and authors regarding the use of this service.
Paper writing (e.g., writing a paper from a thesis/dissertation)	In their correspondence with editors, authors should declare use of such services. Authors could declare the use of such services in their manuscript’s acknowledgments section.	In their correspondence with authors, editors should remind authors of their duty to declare the use of such services.	Publishers should provide a list of best practices for editors and authors regarding the use of this service.
Data fabrication	Authors must not engage in these behaviors and have a duty to report colleagues (or other academics) that do.	Editors must only appeal to (and recruit) competent peer reviewers and should also make the content of peer review reports, manuscript data, and figures publicly available. In cases of confirmed data fabrication before publication, editors should inform the affiliated institutions of the corresponding author. If fabrication is confirmed after publication, editors must issue a retraction notice.	Publishers must make public notices if their journals detect data fabrication after publication of a manuscript. If an investigation is required, an expression of concern can be published.
Providing papers with individuals listed as authors who did not contribute to the paper	Authors must not engage in these behaviors and have a duty to report colleagues (or other academics) that do.	Editors should require a statement from authors that they all made contributions according to a logic of informed trust and in conformity with established guidelines.	Publishers must provide warnings against the use of this practice to authors.
Fake paper production (full service by “paper mills”: authorship trading, intentional plagiarism, fake data and graphics production, communication with journal editors on behalf of authors, etc.)	Authors must not engage in these behaviors and have a duty to report colleagues (or other academics) that do.	Editors must stay up-to-date with these related phenomena and warn authors against their use in their own and other journals.	Publishers must play a proactive role in detecting and reducing this phenomenon.

^1^Several of these clauses could also serve as guidance points for these agents in journals’ instructions for authors

In some cases, such as editing or translation, artificial intelligence (AI) is used to provide assistance, a topic that has become particularly relevant. Since 2022, with the popularization of large language models (LLMs) such as ChatGPT and Google Bard, there is now growing awareness of the rising number of AI-based tools, how they are impacting our societies, and how TPS use AI as ancillary services or assistance in academic publishing. There are many examples in the literature that discuss the nature and operating principles of TPS and their services.

Badenhorst and Xu (2016, p. 4) referred to publishing as a social practice that “includes power inequities and unequal access” and emphasized that an ultra-competitive environment and a “publish or perish” culture drive some authors to rely on TPS so as to become more competitive. They also noted that four traits are necessary to become a fluent and successful academic writer: (i) discourse and analytical skills, (ii) critical literacy, (iii) writing fluency, and (iv) emotional intelligence. We would add individual “knowledge” to this list. In other words, academic writing is not only a skill, but also a kind of personalized art form^4^.

There are several factors that may drive a body of academics towards the use of TPS, such as the requirement to conform to a rigid paper structure^5^ (Badenhorst and Xu 2016) or the adaptation of a certain publishing style (i.e., formatting) (Olson 2020). Other factors reflect non-scientific demands, such as cover letters, that could be seen as a waste of an academics’ time and resources (Teixeira da Silva 2020), exposure to recurrent journal rejections, and the difficulties non-native English speakers face in getting their work published in Anglophone journals. Lines (2016) sees substantive editing as an “insidious form of plagiarism” (p. 368). Although we are not sure how to clearly define “substantive editing,” a given manuscript could fall anywhere on the scale of “no editing,” “minor editing,” “substantial editing,” extending all the way to “pure ghostwriting.”

## The ethics of disclosure

There are two dominant moral and ethical perspectives regarding the use of TPS (both commercial and non-commercial):

1. Consideration of *why* such services are needed and whether they should be provided by the authors’ research institutions rather than by individuals or commercial companies outside of those institutions (Kendall et al. 2016)
2. The importance of reporting/declaring the use of any TPS, regardless of the form or amount of support received, and, more importantly, quantifying the amount of support received

The International Committee of Medical Journal Editors (ICMJE) (ICMJE 2024) already suggests that any reliance on TPS should be acknowledged (Kendall et al. 2016; Teixeira da Silva 2021a). Universities and other academic institutions are closely monitoring developments related to chatbots such as ChatGPT and other LLMs or AI-based tools and their use in teaching and learning.

However, cultural factors must also be considered when assessing whether such assistance qualifies or merits authorship^6^ or whether it should be limited to an acknowledgement (Patience et al. 2019). We have noted that some medical writers are labeled as “ghostwriters” with industry ties and are thus potentially biased (Buck et al. 2023). They may be unsure whether their contribution merits authorship or an acknowledgement (Stocks et al. 2018). Ultimately, the underlying argument—and the common argument throughout this paper—is straightforward: when *intellectual* input or any new content is contributed by a TPS, that assistance must be declared openly, honestly, and transparently. Conversely, hiding the contribution or use of a TPS is a sign of opacity and dishonesty. In essence, an unacknowledged medical writer is tantamount to a ghostwriter (Das and Das 2014). Even if no commercial entity provided technical or linguistic assistance, as is usually the case with TPS, but rather voluntary support, e.g., from a colleague, authors are obligated to offer at least a note of thanks as a formal recognition of this assistance in the Acknowledgements section of a scientific paper (Teixeira da Silva et al. 2023). The Acknowledgements section is therefore the most appropriate section in a paper to indicate the reliance on a TPS (ICMJE 2024), a policy that has been endorsed by a professional conglomerate of professional medical writers (Gertel et al. 2018). Indeed, many authors routinely even thank anonymous reviewers of submitted manuscripts for their valuable feedback, stating (typically) that “*their comments have increased the quality of the paper.*” If reviewers are credited with improving the paper, then why not other TPS?

In our view, the reasons for relying on a TPS can range from valid to invalid. In the camp of *valid* reasons is a genuine lack of technical, linguistic, or other skills to write a paper that will survive the scrutiny of peer review (Tumin and Tobias 2019) and eventual publication. Examples are as follows: (i) genome sequencing, if one does not have a sequencer in one’s laboratory or research institute; (ii) language revision and editing, if one is not a native English speaker; or (iii) statistical consulting. It costs nothing to be clear and honest about the use of a TPS. More importantly, such a statement is an ethical requirement when submitting work for publication to a journal that claims to have and adhere to ethical standards or guidelines, such as those of the ICMJE, although the mandatory nature of these has recently been questioned (Teixeira da Silva 2023).

At the other end of the spectrum is the camp of *invalid* reasons: laziness (i.e., the lack of desire to do or complete a necessary task), embarrassment (i.e., not feeling comfortable or feeling ashamed to disclose the use of a TPS), dishonesty (i.e., not wanting to be honest about the use of a TPS), lack of ability (i.e., a researcher does not have the technical/disciplinary skills to complete the task, in which case those who help should probably also be authors), and outright deception (fake publications produced by paper mills). The camp of invalid reasons may include those with which one can sympathize (on a humanitarian level), but they are still invalid excuses for not reporting the involvement of a TPS. For this reason, such omissions can be considered ethical violations (Lozano 2014).

We also believe that one reason why a reproducibility crisis has emerged in science (Stupple et al. 2019; Draeger et al. 2020; Kapoor and Narayanan 2023) may be related to the undeclared use of TPS. This is partly because the publishing industry has created a culture that allows—and perhaps even implicitly condones—the legitimization of laziness and dishonesty, coupled with a lack of adequate detection methods and low, if any, penalties when ethical violations are detected.

The most egregious example of violations of standing ethics policies are “paper mills,” which we will discuss later. The tide may turn, however, once sufficiently sensitive or robust detection tools become available, such as some simple rules of detection (Sabel et al. 2023). Then, ethical transgressors may be more vulnerable to detection than they were two decades ago, when such tools were not available.

## A new front for fraud? The case of AI-assisted editing

The worrying and growing problem of paper mills (Ritter 2005; Teixeira da Silva 2021b; Byrne et al. 2022; Wykes and Parkinson 2023; Sabel and Seifert 2021), as discussed in the next section, lies at the intersection between the use of human and AI-based services to perform tasks such as editing, writing, and figure generation. Concerns have already been raised about the ability of large language models, such as ChatGPT, to effectively edit text (Tsigaris and Teixeira da Silva 2023; Tsigaris et al. 2023), albeit often imperfectly and occasionally with errors, such as incorrect references, or “hallucinations” (Kim 2023). We are increasingly concerned about how the misuse of LLMs may usher in a new generation of fraud and scientific misconduct, not only by authors, but also by TPS themselves (Hosseini et al. 2023; Kendall and Teixeira da Silva 2024). We also worry about the risk that LLMs pose by undermining the principles of authorship and human scientific endeavor, thereby significantly reducing the integrity of the permanent scientific record. This threat requires publishers, especially the largest and most influential ones (Nishikawa-Pacher 2022), to make serious and effective efforts to detect the use of AI, either by authors or by TPS. Since several for-profit and commercial TPS are core or fundamental to the infrastructure of the publishing industry, and since many of these services use AI to perform or complete their tasks, this may not be compatible with the ethics policies of journals and publishers. Given their commercial conflict of interest, it is questionable whether they will devote the necessary resources to detect fraud with a sufficient level of motivation and due diligence.

As briefly mentioned above, authors may use AI to assist them, e.g., ChatGPT or Google Bard, but TPS themselves are also likely to use or rely on AI. In both cases, but especially in the latter, where companies charge customers for the use of an AI-assisted service, the question arises as to what are the ethical implications of such use. If generative AI tools such as ChatGPT or other LLMs require acknowledgement (Hosseini et al. 2023; Kaebnick et al. 2023; Lingard 2023), then so too should other TPS.

## Paper mills: a debate in the context of TPS and ethical breaches

A phenomenon commonly referred to as “paper mills” is the existence of commercial agencies that are the most notorious example of large-scale dishonesty in the world of science and academic publishing (COPE & STM 2022). Paper mills are for-profit agencies that sell a complete product with partially or completely manipulated, i.e., fabricated, data. Their toolbox for creating fake publications is an assortment of means to cheat, including plagiarized images (Bik et al. 2016), outright data fabrication (Byrne and Christopher 2020; Park et al. 2022), or the use of figures, tables, and text semi-automatically generated by AI (Sabel and Seifert 2021; Sabel et al. 2023). Fake manuscripts are edited or polished by scientifically trained professionals and ghostwriters.

These practices violate the integrity of academic publishing on an industrial scale by producing fake publications (Seifert 2021; Else and Van Noorden 2021; Else 2022; Byrne et al. 2022; Pérez-Neri et al. 2022). Their activities go beyond the degree of opacity mentioned above or the many ways in which content, statistics, ghost or honorary authorships, cherry-picking of data or spin in abstracts can be manipulated (Sabel and Seifert 2021; Sabel et al. 2023).

The emergence of the commercial production of fake publications has undergone an unprecedented development in the last 10 to 15 years. In fact, it has become a billion-dollar industry with more than 1000 paper mills branded as “academic support” agencies. These are located mainly in China, but also in other countries, including, but not limited to, India, Russia, the UK, and the United States of America (Sabel and Seifert 2021; Sabel et al. 2023). Some may collaborate with journals—both legitimate and predatory—and publishers, helping them to solicit manuscripts by sending out mass invitations to find “customers” (scientists) to contribute to “special issues” and conferences, some of which do not even exist. Some paper mills advertise their fictitious “editing services” on the Internet and charge hefty fees to produce and publish fake articles in journals listed in the Science Citation Index Expanded of the Web of Science Core Collection (Christopher 2021; Else 2022; Sabel et al. 2023).

A recent publication estimates the size of the fake publications in the biomedical literature, i.e., as provided by these TPS, i.e., paper mills (Sabel et al. 2023). While the prevalence of fake publications was long thought to be low (1 in 10,000 publications), the number of detected fakes has increased to 1 to 2% of the total volume of published literature, with more recent estimates indicating that approximately 10% of all biomedical publications may be potentially fake and deserve to be red-flagged for further scrutiny (Sabel et al. 2023). The vast majority of such publications originate from authors in China and India, but also from other countries, including Egypt, Russia, and Turkey. The quality of these publications is sufficiently high to remain undetected by most scientists, journal editors, even publishers and readers. Therefore, the detection of such papers before they are published becomes the ultimate goal (Wittau and Seifert 2024).

## An ethical and moral reflection on TPS

At this point, the reader may have appreciated the illicit and undeclared use of TPS, both non-commercial and commercial alike, and the need for transparent disclosure in academic publications. We have also noted how the abuse of trust on a large scale, through the mass commercialization of TPS, in the form of “paper mills”, is leaving a trail of irreversible damage in a significant part of the (mainly) biomedical literature with fabricated articles starting about 15 years ago and now stored in the permanent scientific record. This is happening because the entire industry operates on the premise of “blind faith and trust” (Teixeira da Silva 2022a). In the following sections, we address the issues of trust and trust damage caused by the undeclared use of TPS (non-commercial, commercial, individual, corporate, or by providers of mass-produced fake papers). We consider this from two perspectives: (i) ethical exceptionalism and (ii) false declarations and the moral, ethical, and legal obligations, as well as the correction of omissions.

### Industry-infused ethical exceptionalism?

At this juncture, we consider the possibility of ethical exceptionalism, that is, when the rule applies to everyone but oneself, particularly when that person is part of an elite club of ethicists, policymakers, a unique stratum of service providers, editors, or a member of the commercial publishing industry. In this concept, such individuals seek to excuse, conceal, or otherwise allow dishonest behavior within their own ranks by projecting it as both honest and ethical (Teixeira da Silva 2017). To our knowledge, as documented in the literature, some members of academia or the industry feel that they are entitled to not being thanked or acknowledged for the commercial use of their TPS. They may feel that secrecy is a kind of a right or special privilege, i.e., a classic case of entitlement and ethical exceptionalism, such that hiding behind a curtain of opacity is excused by “authors’ discretion” (Burrough-Boenisch 2019; Matarese and Shashok 2020). This is because acknowledging TPS related to language and editing is not a matter of choice, but rather a matter of valor, i.e., honesty and transparency of declarations, and cannot be attributed to a misalignment of values that may occur when members of academia and industry collaborate (Ingstrup et al. 2021). Turner (2011) has referred to proofreading as an ethical “quagmire.” However, we believe that this would only be the case if such services were not acknowledged as we have argued above. Nonetheless, some find it difficult to distinguish between collaboration and collusion when such assistance is provided, with a range of ethical tolerance and ranking, depending on the individual whose perception is being assessed (e.g., student vs professor) (Kim and LaBianca 2018). One way to eliminate these conflicts was proposed by Kendall et al. (2016), who suggested that “publication consultants should provide an annual return^7^ that details the papers, dissertations and thesis that they have consulted on.”

### False declarations, moral, ethical, and legal obligations, and the correction of omissions

Failure to declare the use of a TPS constitutes an omission or misrepresentation to the journal and ultimately harms the permanent scientific record and the public at large. If authors are not truthful about their intellectual contributions in the papers that they produce, then they are not trustworthy. However, the trustworthiness of researchers is a necessary condition for public society to give its informed consent to the research and publishing enterprise (Coutellec 2020). Hence, the undeclared use of a TPS represents a departure from trustworthiness through negligence, recklessness, or, in more serious cases, deliberate deception (Whitbeck 1995).

These sloppy or deliberate behaviors are not only harmful to the scientific community but also to the individual researchers who are guilty of practicing them. For how can such researchers claim to be intellectually virtuous and rigorous truth-seekers and yet be careless about acknowledging intellectual contributions in the publications they themselves sign? Truthfulness requires not only the veracity of the claims one makes about reality, but also the disclosure of how those claims were made (Heidegger 2002). In other words, we argue that being a virtuous scholar is incompatible with not disclosing TPS.

An unintentional omission can be easily remedied by a correction, but in cases of intentional omission (i.e., deliberate concealment of the use of a TPS) or outright falsification for profit, there should be penalization^8^ through retraction of articles (Parker et al. 2022), as occurs in paper mill production (Candal-Pedreira et al. 2022), or, in severe cases, withdrawal of journals (or even publishers) from the market. If predatory publishers can be criminally prosecuted for making false claims (Manley 2019), then why should authors who use TPS surreptitiously be an exception to the rule, if they rely on linguistic obfuscation to hide their misdeeds (Markowitz and Hancock 2016), or TPS themselves? An estimated 150,000+ papers per year in the biomedical literature may be suspect or fraudulent (Sabel et al. 2023), i.e., produced by the most concerning type of TPS: paper mills. The lack of awareness by the scientific community of the activities of paper mill is the most serious case of underreported TPS. It is the most obvious and serious violation of current ethical guidelines and policies^9^ in the academic publishing industry.

We extend this argument: From the authors’ perspective, the argument is clear. Ultimately, authors make the final decision and either choose to acknowledge such services honestly, openly, and accurately. Or they deviate from ethical norms to cognitively, deliberately, or subconsciously omit such credit for some of the reasons discussed above. But just as TPS arguably have rights and responsibilities regarding intellectual contributions, then to what extent do TPS have a responsibility to ensure that authors who pay for, and use, their services give them credit? We believe that TPS have at least^10^ the moral and ethical obligation to ask authors who use their services to acknowledge their assistance. Moreover, what ethical and legal responsibilities do such TPS have when papers they have edited (or in the case of paper mills, created) and received money violate ethical issues and are subject to retraction?

Therefore, we believe that not only authors, but also publishers such as those listed in Table 1, have a moral obligation and responsibility to make their best efforts to prevent unethical behavior. Moreover, if a TPS discovers that a client has not acknowledged the use of its services, it also has the moral obligation to contact the authors and the editor-in-chief or the editors to request that an erratum be published, to correct this “oversight” (Teixeira da Silva 2022b), which is retroactive. Thus, even if a paper was published 5, 10, or 20 years ago, if it used a TPS but failed to declare it, there is a moral and ethical obligation to correct the published record. Detecting and correcting these false or missing claims in scientific papers is at the heart of one aspect of post-publication peer review (Teixeira da Silva 2022b; Yeo-Teh and Tang 2023). Authors and publishers have a moral obligation to clean up the scientific record and maintain its integrity (Sabel et al. 2023).

However, these proposals would add an additional layer of complexity to the current scientific system, which is built on trust^11^, debate, and mutual inquiry. Moreover, we suspect that in an industry of this size, and with the volume of papers being published each year (in the order of 5 million^12^), such fine-grained controls and corrective and/or retraction actions would be difficult to implement in practice, requiring a concerted effort by learned societies, publishers, and governments. The likelihood of this happening is low.

We ask rhetorically, should publishers, after retracting papers, especially those that are published as open access and having received article processing fees (Teixeira da Silva 2022c; Borrego 2023), put all the blame on authors for the illicit use of TPS (such as paper mills)^13^? Whether (some) publishers are legally “complicit” is a matter for legal experts to decide. If one accepts our arguments in this paper, how can publishers who offer their own TPS, especially those dedicated to linguistic, editing, and technical services (see Table 1), not insist that their clients acknowledge this contribution? If nothing else, it would be another marketing arm for their revenue-generating services. We also note that publishers benefit from TPS, financially or otherwise (Teixeira da Silva and Vuong 2021; Butler et al. 2022).

## Conclusion

The commercial publishing industry relies heavily on TPS to support its infrastructure for providing a wide range of services to authors. If authors were more self-sufficient, there would be less need for TPS. Yet, we recognize that not all authors are self-sufficient and thus require TPS. However, reliance on TPS is not always for good reasons, such as a lack of skills, rather than a genuine, legitimate need. Many, though certainly not all, TPS have become part of a “cottage industry,” selling services for profit with the (not easily quantifiable) risk of undermining the basic principles of honesty, authority, and ethics. TPS that offer unethical services are probably fully aware that their actions are unethical, yet they feel no shame in offering their services on the Internet to make a large financial profit. More importantly, authors who knowingly use illegitimate services and do not declare their use of TPS are equally shameless and engage in dishonest and unethical behavior by concealing their use of such services in order to gain recognition, promotions, or salary increases. Whatever the reason—such as personal shame or the industry-induced culture of “publish-and-perish” (Guraya et al. 2016)—there are no excuses (other than the desire to conceal) for not disclosing (hiding) the use of TPS. Therefore, we suggest that journal instructions to authors include a number of aspects that would provide clear and unambiguous guidance to prospective authors, while holding both authors (for lack of disclosure) and editors, journals, and publishers (for lack of detection or verification) accountable (Table 2). Perhaps the explosion of paper mill-derived publications, which are widely contaminating the scientific literature and databases, can serve as an impetus for a no-tolerance crackdown on dishonest authors who misrepresent (i.e., fail to disclose) their use of TPS. This dishonesty is a violation of the ethics codes of the most scientific journals, and it raises the question of whether the publisher-associated TPS should also be scrutinized as well. It remains to be seen whether TPS and academic publishers, in this large crisis of scientific publishing, will commit themselves to the fundamental values of scientists, to search for new and “true” knowledge and discovery rather than being just guided by the financial interests of their shareholders.

## Data Availability

No datasets were generated or analysed during the current study.

## Footnotes

1 Preprints are complex in their own right because they are documents that are open to public comment. It is unclear how comments from the public, other scientists, and anonymous sources should be acknowledged.

2 Detection here means the ability to discern whether declarations in papers are true or not. Such statements would be related to the use of TPS or other tools/assistance and need to be noted in the methods section of a paper, or in its Acknowledgements.

3 It is not clear to us precisely what such individuals actually do or what qualifications they may have.

4 We note here that the permeability of intellectual contributions to a paper shows that the individual-only view of “authorship-as-art” should not be overemphasized.

5 Such as the IMRD (Introduction, Materials and Methods, Results, and Discussion) format (Sollaci & Perreira, 2004).

6 The purpose of this paper is not to enter the realm of the debate about what constitutes authorship.

7 They should make a public copy available on their websites.

8 We note here that the concept of using retractions not as a form of correcting the literature (Teixeira da Silva 2022a), but as a form of academic penalization/punishment, may be in direct conflict with a core principle of the Committee on Publication Ethics (COPE) regarding retractions: “The main purpose of retractions is to correct the literature and ensure its integrity rather than to punish authors who misbehave” (Wager et al. 2009).

9 Of relevance is the often linguistic obfuscation caused by the terms “guidelines” and “recommendations”, as used by the two most popular sets of publishing ethics policies in the global academic publishing industry (Teixeira da Silva 2023).

10 There is also likely to be a legal obligation in the sense that legal statements/declarations (i.e., promises and affirmations/assurances) are made by authors upon submission to ICMJE- and COPE-compliant journals regarding honesty, adherence to journal policies, fulfillment of ethical obligations, and so on.

11 The culture is mostly based on “blind trust” (Teixeira da Silva 2022a), which needs to be reformed.

12 https://www.scimagojr.com/countryrank.php?year=2023

13 Here is a recent example: https://retractionwatch.com/2023/10/31/guest-post-a-look-behind-the-scenes-of-bulk-retractions-from-sage/

## Declarations

### Disclaimer

The views expressed in this paper are solely those of the authors and should not be construed as the views of the organizations for which they work.
